# High risk histopathological factors in retinoblastoma in upfront enucleated eyes: An experience from a tertiary care centre of Pakistan

**DOI:** 10.12669/pjms.38.ICON-2022.5787

**Published:** 2022-01

**Authors:** Nausheen Yaqoob, Salima Mansoor, Kanwal Aftab, Bushra Kaleem, Ahmer Hamid, Saba Jamal

**Affiliations:** 1 Nausheen Yaqoob, FCPS, Department of Histopathology, Indus Hospital & Health Network, Karachi, Pakistan; 2 Salima Mansoor, MBBS, Department of Histopathology, Indus Hospital & Health Network, Karachi, Pakistan; 3 Kanwal Aftab, FCPS, Department of Histopathology, Indus Hospital & Health Network, Karachi, Pakistan; 4 Bushra Kaleem, MPhil (Haematology) Indus Health Research Center, Indus Hospital & Health Network, Karachi, Pakistan; 5 Ahmer Hamid, FCPS Department of Paediatric Oncology, Indus Hospital & Health Network, Karachi, Pakistan; 6 Saba Jamal Diplomate American Board of Haematology, Diplomate American Board of Anatomic and Clinical Pathology, Department of Histopathology, Indus Hospital & Health Network, Karachi, Pakistan

**Keywords:** Retinoblastoma, intraocular malignancy, high-risk features

## Abstract

**Background & Objectives:**

The assessment of histopathological risk factors (HRFs) in retinoblastoma in upfront enucleated eyes is important in deciding treatment protocols. Limited data is available from the developing countries as very few studies were conducted on retinoblastoma. The study aims to report this data from Pakistan.

**Methods:**

This cross-sectional study included treatment naïve retinoblastoma patients who underwent upfront enucleation between 2017 to 2021. Various tumor characteristics i.e. laterality, size, histologic grade, anaplasia grade, growth pattern, extent and length of optic nerve invasion, pathologic staging, tumor involvement of ocular structures were assessed. High-risk factors such as involvement of anterior chamber, choroidal, scleral, extrascleral, and optic nerve were also noted.

**Results:**

A total number of 54 patients were enrolled, out of which 53.7% were females while remaining were males. Median age at presentation was 24 months. Unilateral tumor was seen in 92.6% cases. Most frequent histologic grade was G2 (64.7%) and moderate anaplasia was observed in 59.2% cases. Vitreous involvement was seen in (86.5%). Pathologic staging of most of the tumors was pT1 (39.2%). Assessment of high-risk factors revealed that optic nerve involvement (35.1%) was the most common finding with retrolaminar tumor invasion seen in 75% cases. Choroidal invasion (≤3mm) was seen in 55.6% of patients. Limited involvement of anterior chamber (3.8%), sclera (7.4%), and extrascleral (3.8%) tissue was also observed.

**Conclusion:**

The presence of high risk histopathological factors in enucleated eyes diagnosed with retinoblastoma are known to have a profound impact on the risk stratification as well as decision of future treatment plan.

## INTRODUCTION

Retinoblastoma (Rb) is the most common primary intraocular malignancy of the neurosensory retina accounting for approximately 3% of childhood malignancies. Its incidence is between 1:17,000 - 1:20,000 live births and approximately 7000-8000 cases are reported per annum.^1^ It exclusively affects infants and young children and shows no significant race or gender predilection. According to World Health Organization (WHO), 66% cases are detected before 24 months whereas approximately 95% cases are seen before five years of age.^2^ Local literature reports the incidence to be 4 and 2.4 in 100,000 children before age five and ten respectively.^3^ In Asia-Pacific region, India reports highest number of Rb cases while Pakistan ranks sixth.^4^

Retinoblastoma is primarily a clinical diagnosis and histological evaluation of enucleated eyes for the presence of high risk factors is important in deciding further treatment including adjuvant chemotherapy. Histological presence of high risk factors (HRFs) can predict local recurrence ,distant metastases, tumor progression and overall prognosis. HRFs such as choroidal, scleral, extrascleral, and optic nerve invasion (posterior to lamina cribrosa or up to the cut end of the optic nerve) are predictors of metastases.^5^

Incidence of HRFs and systemic metastases is reported to be less in high income countries,^6^-^8^ the reason being early presentation, diagnosis and treatment as compared to advanced-stage disease presentation in developing countries due to lack of education, low socioeconomic status and lack of access to health care facilities.^9^

Treatment of retinoblastoma depends on various factors including laterality and stage of disease. Chemotherapy along with local therapies like brachytherapy, cryotherapy and laser can be used to salvage eyes with early intraocular disease.^10^

Despite of advancement seen in early diagnosis and treatment of retinoblastoma, it’s management still remains a challenge in developing countries. This study assesses the frequency of HRFs in enucleated eyes which would be helpful in deciding future management of the patients.

## METHODS

A cross-sectional observational analysis was performed on enucleated eye specimens of all patients who had a clinical diagnosis of intraocular retinoblastoma presenting to our institute between September 2017 and February 2021.

### Ethical Approval:

The study participants were enrolled after approval from the ethics review board of the institute (IHHN_IRB_2021_03_007) in accordance to the guidelines of Declaration of Helsinki and an exemption was provided due to retrospective nature of the study. Treatment naïve retinoblastoma patients <18 years and of either gender were included in the study.

Patient demographics included age and gender. Tumor characteristics included laterality, size of tumor, histologic grade, anaplasia grade, growth pattern (exophytic or endophytic), extent of optic nerve (ON) invasion, pathologic staging, tumor involvement of ocular structures, and additional findings (calcification, necrosis, inflammation, hemorrhage). Histologically, well differentiated tumors (G1) contained areas of retinocytoma (neuronal differentiation or fluerettes), moderately differentiated tumors were tumors with many Flexner-Wintersteiner or Homer-Wright rosettes (G2), or occasional Flexner-Wintersteiner or Homer-Wright rosettes (G3), while poorly differentiated (G4) tumors lacked any evidence of rosettes formation and/or extensive anaplasia (>50%). Endophytic growth pattern indicated growth from the inner retinal surface into the vitreous cavity while exophytic tumors grew primarily from the outer surface of the retina into the subretinal space toward the choroid. Combined growth pattern exhibited features of both endophytic and exophytic growth.

### High-risk factors:

**Fig.1:**
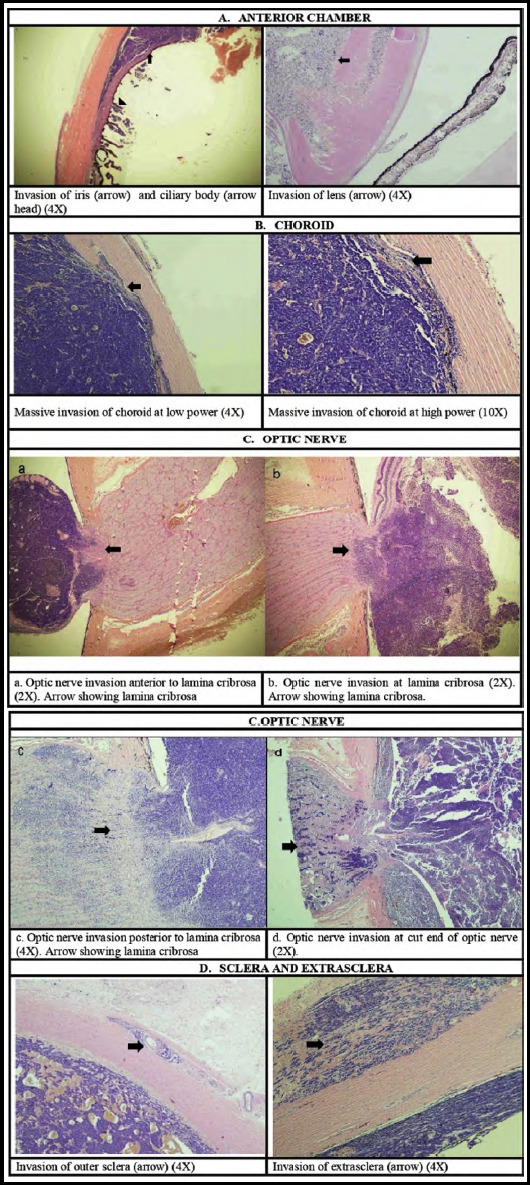


1. Anterior chamber invasion involving iris, ciliary body (Fig.1A)
2. Choroidal invasion (Fig.1B):
  a. Massive: >3 mm in diameter and/or with the full thickness of choroidal involvement.
  b. Minimal: ≤3 mm in diameter and with partial-thickness of choroidal involvement.
3. The optic nerve invasion posterior to lamina cribrosa and to the transection line of the nerve (Fig.1C).
4. Scleral or extrascleral involvement (Fig.1D).

### Statistical Analysis:

The statistical analysis was performed using SPSS version 24.0. Continuous parameters such as age and size of tumor were represented by median (interquartile range) or mean ± SD depending on the normality while categorical parameters such as gender, laterality, histologic grade, anaplasia grade, growth pattern, extent of optic nerve invasion, pathologic staging, tumor involvement of ocular structures and additional findings were represented in proportions. The categorical data were compared using Chi-square (Fisher exact) test as per the need. A p-value of <0.05 was considered statistically significant.

## RESULTS

The study included 54 patients of retinoblastoma during the study period. The median age at presentation was 24 months (IQR: 18-36 months). Female preponderance i.e. 53.7% was observed. Review of enucleated eyes with retinoblastoma revealed following histopathological features and high-risk factors (Table-I). Comparison of various factors with degree of differentiation of tumor is displayed in (Table-II). Certain ocular features could not be assessed in few cases due to non-visualisation of these structures as a result of extensive tumor necrosis.

**Table I:** Histopathological features of retinoblastoma

Variables	Values
**Laterality:**	
Unilateral, n (%)	50 (92.6)
Bilateral, n (%)	4 (7.4)
**Tumor size, cm**	
Median (IQR)	1.7 (1.5 - 2.0)
**Histologic grade (n=51)**	
Well-differentiated (G1), n (%)	4 (9.8)
Moderately differentiated (G2), n (%)	34 (64.7)
Moderately differentiated (G3), n (%)	11 (21.6)
Poorly differentiated (G4), n (%)	2 (3.9)
Could not be determined, n (%)	3
**Anaplasia grade (n=49)**	
Mild, n (%)	20 (40.8)
Moderate, n (%)	29 (59.2)
Severe, n (%)	0
**Growth pattern (n=53)**	
Endophytic, n (%)	41 (77.4)
Exophytic, n (%)	7 (13.2)
Combined endophytic and exophytic, n (%)	5 (9.4)
**Tumor involvement of other ocular structures:**	
Vitreous, n (%)	45/52 (86.5)
Optic nerve head, n (%)	24/31 (77.4)
Sub-retinal pigment epithelial space, n (%)	17/27 (63.0)
Sub-retinal space, n (%)	29/47 (61.7)
Optic disc, n (%)	8/20 (40.0)
Cornea, n (%)	1/53 (3.8)
**Optic nerve invasion (n=36)**	
Anterior to lamina criborisa, n (%)	15 (41.6)
At lamina criborisa, n (%)	5 (13.9)
Posterior to lamina cribrosa, n (%)	15 (41.6)
Up to transection line of ON, n (%)	1 (2.8)
**Pathologic staging (n=51)**	
pT1, n (%)	20 (39.2)
pT2, n (%)	1 (2.0)
pT2a, n (%)	12 (23.5)
pT3, n (%)	4 (7.4)
pT3b, n (%)	10 (19.6)
pT3d, n (%)	1 (2.0)
pT4, n (%)	2 (3.9).
**Additional findings:**	
Necrosis, n (%)	44/50 (88.0)
Calcification, n (%)	32/51 (62.7)
Inflammation, n (%)	21/54 (38.8)
Haemorrhage, n (%)	7/54 (12.9)
Retinal detachment, n (%)	2/54 (3.7)
**High-risk factors**	
**Anterior segment structures involvement (n=53)**	
Iris, n (%)	2 (3.8%)
Ciliary body, n (%)	2 (3.8%)
**Choroidal invasion (n=18)**	
≤3mm, n (%)	10 (55.6)
>3mm, n (%)	8 (44.4)
**Extent of optic nerve invasion (n=20)**	
Posterior to lamina cribrosa, n (%)	15 (75.0)
Up to transection line of the nerve, n (%)	1 (5.0)
Scleral invasion, n (%)	4/54 (7.4)
Extrascleral invasion, n (%)	1/54 (1.9)

**Table II:** Univariate analysis of variables with degree of differentiation of tumor

Degree of tumor differentiation	Number of HRF					Presence of HRF			Laterality			Age categories		

	No HRF	1 HRF	2 HRF	3 HRF	p-value	No HRF	>=1 HRF	p-value	Unilateral	Bilateral	p-value	<24 months	>=24 months	p-value
Well differentiated (G1); n=4	3 (75)	1 (25)	0	0	0.640	3 (75)	1 (25)	1.000	3 (75)	1 (25)	0.329	3 (75)	1 (25)	0.192
Moderately differentiated (G2); n=33	21 (61.8)	7 (20.6)	6 (17.6)	0		21 (61.8)	13 (38.2)		31 (91.2)	3 (8.8)		21 (61.8)	13 (38.2)	
Moderately differentiated (G3); n=11	7 (63.6)	2 (18.2)	1 (9.1)	1 (9.1)		7 (63.6)	4 (36.4)		11 (100)	0		4 (36.4)	7 (63.6)	
Poorly differentiated (G4); n=2	1 (50)	0	1 (50)	0		1 (50)	1 (50)		2 (100)	0		0	2 (100)	

## DISCUSSION

Enucleation is one of the oldest form of treatment for retinoblastoma particularly in advanced cases^9^ and considered as the best treatment option for patients with unilateral disease and non-salvageable vision. Enucleated eyeball is always assessed for the presence of HRFs. Presence of HRFs warrants the risk of secondary orbital recurrence as well as systemic metastasis, and thus there is a strong indication of adjuvant treatment comprising of chemotherapy and/or External Beam Radiation Therapy (EBRT) which resulted in risk reduction of metastasis to 4% in comparison to 24% who did not receive the treatment according to Honovar et al.^11^

Histopathological features of retinoblastoma have been the focus of many studies.^6^-^8^,^12^,^13^ The present study assessed the histopathological features of retinoblastoma with an emphasis on the high-risk factors. Mean age of presentation in the present study was 29.15 ±18.20 months (Median – 24; IQR-18.0 – 36.0 months) which was similar to findings of other regional studies by Zia et al^14^, and Gupta et al.^12^ also reported that age at presentation greater than 24 months was a predictor for HRFs. In our study, HRFs were noted in 24.0% patients who presented at >24 months of age.

The present study reports presence of well-differentiated tumors in patients who presented at age <24 months in comparison to poorly differentiated tumors who presented in older age group (Table-II). Kashyap et al.^15^ also reported poorly differentiated tumors in patinets presenting at an age of >24 months. Present study also reported presence of HRFs in tumors which showed poor differentiation (Table-II) This was similar to another study in which multiple HRFs were observed in poorly differentiated tumors (26%) in comparison to well-differentiated tumors (6.7%).^15^

The HRFs include optic nerve invasion (posterior to lamina cribrosa or to the cut end of the nerve), choroidal, scleral, and extrascleral involvement.^5^ The number of patients with ≥1 HRFs in the present study was 35.2% which was similar to the prevalence reported by Kaliki et al.^13^ i.e. 38%. Our study reported 1 HRF and >1 HRF in 18.5% and 16.7% patients respectively which was in concordance to a study by Kashyap et al.^15^ which reported 18.7% and 22.7% patients for the same. These HRFs have been reported to have a varying prevalence i.e. 6%-28% for invasion of optic nerve posterior to lamina cribrosa and optic nerve to the resection line, 12%-42% for choroidal involvement and 8%-15% for scleral and extrascleral spread.^9^ Comparison of the frequency of HRFs observed in our study was compared with other studies from nearly two decades as displayed in Table-III.

**Table III:** Comparison of studies reporting the prevalence of high-risk factors in retinoblastoma.

Study	Anterior chamber		Choroidal invasion, n (%)	Scleral invasion, n (%)	Extrascleral invasion, n (%)	Retrolaminar ON involvement, n (%)	ON to cut end involvement, n (%)

	Iris, n (%)	Ciliary body, n (%)					
Biswas et al.^7^ (n==232; 2003)	NA	NA	51 (21.9)	NA	NA	13 (5.6)	NA
Orellana et al.^8^ (n=101;2009)	NA	NA	42 (41.5)	9 (8.9)	10 (9.9)	40 (39.6)	NA
Gupta et al.^12^ (n=142; 2009)	10 (7)	13 (9)	57 (40.1)	13 (9.1)	9 (6.3)	24 (16.9)	11 (7.7)
Kashyap et al.^15^ (n=609; 2012)	65 (10.7)	NA	150 (24.6)	83 (13.7)	25 (4.1)	98 (16.1)	45 (7.4)
Kashyap et al.^17^ (n=326; 2012)	29 (9)	23 (7)	71 (21.2)	28 (9)	11 (3.4)	54 (17)	18 (5.5)
Yousef et al.^18^ (n=50; 2014)	NA	3 (6)	9 (18)	NA	NA	7 (14)	NA
Rao et al.^19^ (n=17; 2014)	5 (29.4)	5 (29.4)	10 (58.8)	5 (29.4)	2 (11.8)	NA	4 (23.5)
Kaliki et al.^16^ (n=403; 145 cases, 285 controls; 2015)	12 (8)	17 (12)	96 (66)	20 (14)	8 (6)	71 (49)	3 (2)
Kaliki et al.^9^ (331 Indians/193 Ameircans; 2018)	11 (3.3) / 9 (5)	14 (4.2) /6 (3)	57 (17) / 12 (5)	19 (5.7) / 3 (2)	7 (2.1) / 2 (1)	56 (16.9) / 22 (11)	2 (0.6) / 1 (0.5)
Yahaya et al.^20^ (28) (n=234; 2019)	37 (21.5)		47 (27.3)	39 (22.7)	NA	67 (28.5)	NA
Kaliki et al.^13^ (n=616; 2020)	28 (4.5)	29 (4.7)	120 (19.5)	30 (4.9)	10 (1.6)	103 (16.7)	11 (1.8)
Present study (n=54; 2021)	2 (3.7)	2 (3.7)	18 (33.3)	4 (7.4)	1 (1.9)	15 (41.7)	1 (2.8)

ON-Optic nerve; NA-Not available.

The extent of optic nerve invasion is one of the HRFs with incidence of metastasis being reprorted in 12%-42%^9^ cases involving posterior to lamina cribrosa and up to 41%-78% involving transection line.^11^ In present study, 66.7% of eyes had optic nerve involvement with variable degree of invasion i.e. both anterior and posterior to lamina cribrosa. An unusual finding in the present study was a very high prevalence (41.7%) of involvement of optic nerve posterior to lamina cribrosa which was similar to the frequency reported by Kaliki et al. (49%)^16^ and Orellana et al. (39.6%)^8^ (Table-III). The higher frequency of optic nerve involvement were all reported from low middle income countries and thus could be attributed to the fact that patients present at advanced stage of disease due to lack of access to health care facilities while another reason could be lack of awareness among parents and health care providers at primary health care level.

In present study, frequency of choroidal involvement was 33.3% out of which massive invasion of choroid was present in 44% cases. This observation of massive choroidal involvement was similar to the frequency reported by Orellana et al.^8^ i.e. 41.3% but was much higher to an Indian study which reported 27.5% cases with choroidal invasion.^9^ Choroidal involvement is considered to be a high risk factor for metastases particularly if seen in association with optic nerve involvement.^9^

The involvement of anterior chamber structures i.e. iris and ciliary body also constitutes a HRF. The present study reported the involvement of iris and ciliary body in 3.7% cases each. This finding was found to be similar to the reported frequencies of ciliary body (4.7%) and iris (4.7%) involvement in an Indian study by Kaliki et al.^13^

Scleral invasion is the invasion of the tumor beyond the choroid. Current study reported the frequency of this HRF in 7.4% cases which was relatively closer to the frequencies reported by various Indian studies.^8^,^12^,^17^ Extrascleral invasion was seen in 1.9% of the patients which was almost the same as the frequency i.e. 1.6% reported by Kaliki et al.^13^ as well in the Indian patients cohort which compared the occurrence of HRFs between American and Indian retinoblastoma patients.^9^

### Limitations:

The short study span and small sample size may be considered as limitations of our study. Presence of high-risk factors in these patients was not further evaluated by follow-up to assess the impact of their presence on disease progression which warrants further research to analyse outcome in context of presence of HRFs.

## CONCLUSION

In conclusion, this is a retrospective data analysis of high risk histopathologic factors in eyes diagnosed with retinoblastoma and treated with primary enucleation from a low middle income country. As our center is a major tertiary care referral hospital, our results might be highly representative of high risk factors in retinoblastoma in our population. The incidence of HRFs has declined over time in studies from developed countries because of early presentation and diagnosis. However, the same is not seen in studies from the developing world. Late presentation of patients, age >24months, and with more advanced disease result in poor outcome in retinoblastoma.

### Authors’ Contribution:

**NY:** Substantial contribution towards the concept of the study, revised it critically for important intellectual content;

**SM:** Drafted the work, revised it critically for important intellectual content;

**KA, SAH, SJ:** Revised it critically for important intellectual content;

**BK:** Substantially contributed towards data acquisition, analysis, and interpretation; drafted the work;

All authors approved the final version and also agreed to be accountable for all aspects of the work in ensuring that questions related to the accuracy or integrity of any part of the work are appropriately investigated and resolved.
